# Using event logs to observe interactions with electronic health records: an updated scoping review shows increasing use of vendor-derived measures

**DOI:** 10.1093/jamia/ocac177

**Published:** 2022-09-29

**Authors:** Adam Rule, Edward R Melnick, Nate C Apathy

**Affiliations:** Information School, University of Wisconsin–Madison, Madison, Wisconsin, USA; Emergency Medicine, Yale School of Medicine, New Haven, Connecticut, USA; Biostatistics (Health Informatics), Yale School of Public Health, New Haven, Connecticut, USA; MedStar Health National Center for Human Factors in Healthcare, MedStar Health Research Institute, District of Columbia, Washington, USA; Regenstrief Institute, Indianapolis, Indiana, USA

**Keywords:** electronic health record, audit log, event log

## Abstract

**Objective:**

The aim of this article is to compare the aims, measures, methods, limitations, and scope of studies that employ vendor-derived and investigator-derived measures of electronic health record (EHR) use, and to assess measure consistency across studies.

**Materials and Methods:**

We searched PubMed for articles published between July 2019 and December 2021 that employed measures of EHR use derived from EHR event logs. We coded the aims, measures, methods, limitations, and scope of each article and compared articles employing vendor-derived and investigator-derived measures.

**Results:**

One hundred and two articles met inclusion criteria; 40 employed vendor-derived measures, 61 employed investigator-derived measures, and 1 employed both. Studies employing vendor-derived measures were more likely than those employing investigator-derived measures to observe EHR use only in ambulatory settings (83% vs 48%, *P* = .002) and only by physicians or advanced practice providers (100% vs 54% of studies, *P* < .001). Studies employing vendor-derived measures were also more likely to measure durations of EHR use (*P* < .001 for 6 different activities), but definitions of measures such as time outside scheduled hours varied widely. Eight articles reported measure validation. The reported limitations of vendor-derived measures included measure transparency and availability for certain clinical settings and roles.

**Discussion:**

Vendor-derived measures are increasingly used to study EHR use, but only by certain clinical roles. Although poorly validated and variously defined, both vendor- and investigator-derived measures of EHR time are widely reported.

**Conclusion:**

The number of studies using event logs to observe EHR use continues to grow, but with inconsistent measure definitions and significant differences between studies that employ vendor-derived and investigator-derived measures.

## INTRODUCTION

Healthcare operations, policymaking, and research increasingly involve measuring how clinicians interact with electronic health records (EHRs). From measuring the impact of policy and pandemic on EHR use,^1–3^ to identifying clinicians with the greatest documentation burden,^4^^,^^5^ to investigating the links between EHR use and burnout,^6–9^ there is a growing demand for fast, accurate, and cheap ways to measure EHR use. Measuring EHR use at the scale needed to inform policy and practice is difficult. Direct observation yields rich contextual data but is slow, expensive, and prone to observation bias.^10^ Surveys are cheaper and easier to scale but prone to several types of reporting bias including acquiescence and social desirability bias. Both methods also suffer from selection bias. To avoid these issues, investigators increasingly rely on event logs to observe EHR use.^11^^,^^12^

EHR event logs are a diverse set of computer-generated files that track EHR operation and use. These logs track system events, which may be prompted by user actions (e.g., clicks that send a message or open a note) or events internal to the EHR (e.g., server responses to data requests). All certified EHRs are required to maintain at least 1 event log to support audits of record access; an “audit log” tracking when users view, edit, or print any portion of a patient record. Many EHRs also maintain additional event logs tracking specific user activities such as note writing or inbox messaging. For example, many EHRs maintain logs tracking how text templates are used to write documents such as notes (e.g., who invoked which templates to write which documents and when). Together, these diverse event logs enable investigators to constantly and passively collect data on EHR use without the costs or biases of surveys or direct observation.^12^

However, raw EHR event logs contain tremendous amounts of data. A year of raw event logs for a single institution can reach 100s of Gigabytes, making them difficult to store, access, and analyze. These logs must also be heavily processed to derive meaningful measures such as the time clinicians spend using the EHR. More concretely, creating time-based measures requires making nontrivial decisions about how to map individual actions to clinical activities (i.e., was the clinician doing chart review, or responding to a patient message while viewing those labs) and how to handle gaps between recorded actions (i.e., was the clinician reading a note for 3 min, or had they stepped away from the EHR).

Several EHR vendors now automatically derive measures of EHR use from event logs—typically summarized by week or month—and present them to administrators in interactive dashboards.^13^ These vendor-derived measures are widely used in healthcare operations and increasingly used in research.^14^ By automating the labor-intensive process of turning raw event logs into meaningful measures, vendor-provided measures increase the number of individuals and organizations who can use event logs to observe EHR use and enable more consistent measurement of EHR use across organizations. However, researchers and administrators have noted several limitations of vendor-derived measures including opaque measure definitions, measures changing without notice, a lack of validation, inconsistency across vendors, and misalignment with clinical workflows (e.g., in definitions of scheduled working hours).^15–19^

We previously reviewed the literature published before July 2019 in which investigators derived their own measures of EHR use from EHR audit logs.^11^ We found investigators created a diverse array of measures from audit logs, but that many of these measures had not been validated, and that variability in measure definition hindered the comparison of results across studies. The increasing use of vendor-derived measures—which are derived from both audit logs and other types of event logs—raises new questions about the state and scope of EHR event log research. Specifically, there is a need to understand (1) how often vendor-provided measures of EHR use are used in research, (2) differences in the kinds of research conducted with vendor and investigator-derived measures which may reflect an affinity or bias in the research conducted with each type of measure, and (3) whether a consistent set of measures is being used so that results can be synthesized and compared across studies.

### Objective

The objective of this scoping review is to compare the aims, measures, methods, limitations, and scope of studies that employ vendor-derived and investigator-derived measures of EHR use, and to assess measure consistency across studies.

## MATERIALS AND METHODS

We followed PRISMA guidelines for this scoping review and registered our protocol with the Open Science Framework (https://osf.io/h6d7j). We included peer-reviewed articles which (1) reported original research, (2) analyzed measures derived from EHR event logs, and (3) were published between July 2019 and December 2021. We restricted our search to this period to avoid overlapping with the prior review,^11^ or conflating differences between studies with historical trends. We excluded articles based on clinical decision support (CDS) logs as the CDS literature has been thoroughly reviewed elsewhere.^20–22^

As in the prior review,^11^ we identified relevant articles by combining a list of known articles with those obtained by querying PubMed and citation search. To select query keywords, we observed how relevant literature described EHR event logs and vendor-derived measures as there are no MeSH terms for these concepts. We hand-selected 48 articles published after June 2019 and extracted relevant keywords from each article’s title and abstract. We combined these keywords with those used in the prior review to create an updated query for EHR event log research.^11^ See the Supplementary eMethods in the supplement for the full query and inclusion criteria.

**Figure 1.**
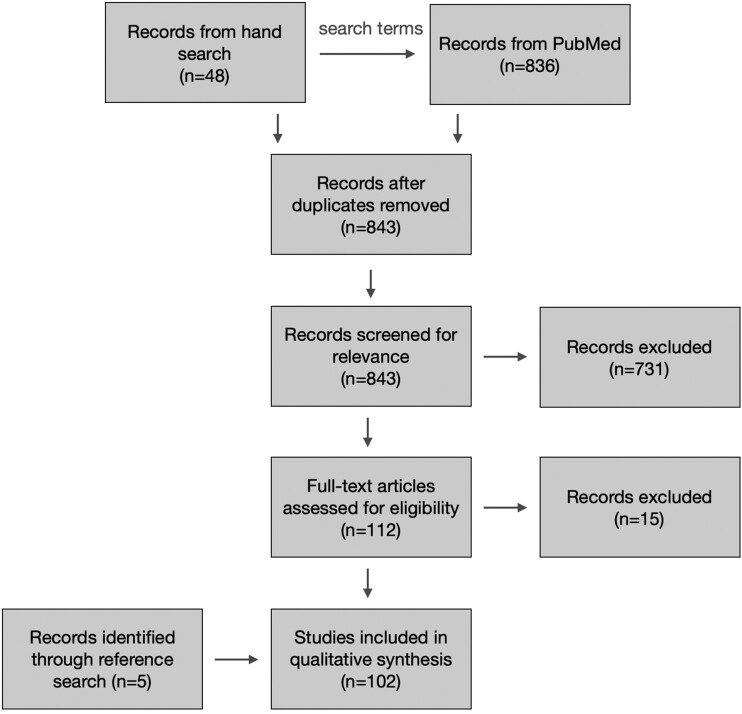
Article search and review process.

The PubMed query returned 836 articles which, together with the 48 seed articles, yielded 843 unique articles for review (Figure 1). Two authors with extensive experience analyzing EHR event logs (NCA and AR) reviewed the title and abstract of each article and identified 112 articles for further review through consensus. Upon reviewing the full text of each article, 15 articles were removed from review, while 5 articles were added through backward and forward citation search, yielding 102 articles for data abstraction.

We used an updated version of the coding scheme of study aims, measures, methods, and scope employed in the prior review for data abstraction.^11^ While the prior coding scheme identified general measures of EHR use (e.g., counts of actions, durations of time), we updated the scheme to include binary codes for whether each article reported 9 more specific measures of EHR use. Seven of these more specific measures were based on those proposed by Sinsky et al^23^ (i.e., total EHR time, time in notes, time in inbox, time in orders, time outside scheduled hours, teamwork for orders, undivided attention). Two additional measures emerged from the reviewed literature (i.e., time in chart review, inbox message volume). We also added codes for whether the study analyzed investigator-derived or vendor-derived measures, and whether investigator-derived measures were derived from audit logs or other EHR event logs (e.g., text template logs, inbox messaging logs). To assess measure consistency, we coded the denominators used to normalize durations of EHR time (e.g., time per day, per appointment) and extracted the method used to determine if the EHR was actively being used. To validate and refine the coding scheme, 2 authors (NCA and AR) independently coded 10 articles, achieving high inter-rater reliability (i.e., Cohen’s Kappa of 0.69). They discussed the source of coding differences and updated codebook definitions accordingly. A single author (AR) then coded the remaining articles.

We used Fisher’s exact test to identify significant differences in the aims, measures, methods, limitations, and scope of studies employing vendor-derived and investigator-derived measures and Mann-Whitney U tests to compare study size. We set the threshold for significance at *P* < .05. There are no new data associated with this article.

## RESULTS

### Number of studies

**Figure 2.**
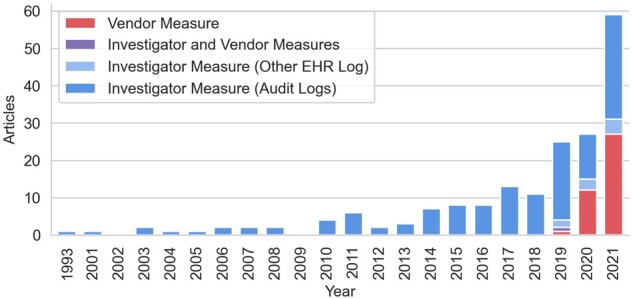
Sources of log-based data used in EHR research. Figure combines articles from the current review with a prior review of EHR audit log literature published before July 2019. EHR: electronic health record.

Of the 102 articles included in this review, 40 employed vendor-derived measures of EHR use,^2–4^^,^^6^^,^^19^^,^^24–58^ 61 employed investigator-derived measures ,^7^^,^^59–118^ and 1 employed both (which we exclude from the comparisons that follow).^119^ See Supplementary eTable 1 for a summary of article details. Of the 62 articles that employed investigator-derived measures, 53 derived measures from audit logs,^7^^,^^59–109^^,^^119^ and 9 derived measures exclusively from other EHR event logs (e.g., inbox messaging logs).^110–118^Figure 2 shows annual counts of log-based research as identified in the current review and the prior review of audit log research.^11^ While the current review includes a broader range of EHR logs and log-based measures, 2 post hoc analyses validate the apparent increase in log-based research. First, the prior review identified 33 studies published in the 2½ years before July 2019 in which investigators derived their own measures of EHR use from audit logs.^11^ This review identified 53 such studies published in the 2½ years that followed, a 61% increase in the rate of audit log research. Second, when we extended our search query to include articles published before July 2019, we only identified 4 additional articles which analyzed vendor-derived measures, the earliest of which was published in 2018.^120–123^

### Differences in study scope and size

**Figure 3.**
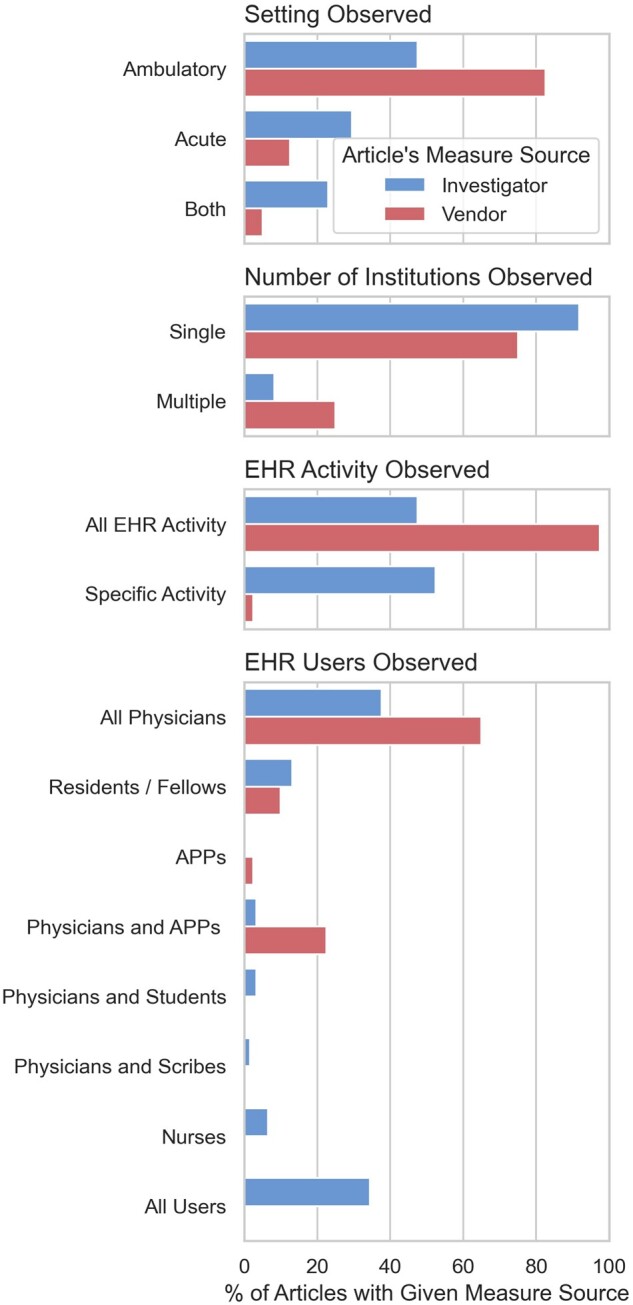
Article scope, by source of measures reported in article.

We observed several significant differences in the scope of studies employing vendor and investigator-derived measures (Figure 3 and Supplementary eTable 2). First, studies employing vendor and investigator-derived measures observed different clinical settings (*P* = .002). While most vendor-measure studies (83%) observed EHR use exclusively in ambulatory settings,^2–4^^,^^6^^,^^19^^,^^24–51^ half of investigator-measure studies (52%) observed at least some EHR use in acute (i.e., inpatient or emergent) care.^78–109^ Studies employing vendor- and investigator-derived measures also included different participants (*P* < .001). While vendor-measure studies only ever included physicians or advanced practice providers (APPs), a third of investigator-measure studies (34%) included *all* EHR users who performed the observed activity,^73–76^^,^^84–90^^,^^103–108^^,^^115–118^ and another 11% specifically included nurses, medical students, or scribes.^77^^,^^91–95^^,^^109^ Vendor-measure studies were more likely to include data from multiple institutions (25% vs 8% of studies, *P* = .025) and to observe overall EHR use, rather than only collect data on a specific activity such as note writing or inbox management (98% vs 48% of studies, *P* < .001). The median number of participants (201 vs 172, *P* = .08) and organizations observed (1 vs 1, *P* = .42) were not significantly different between vendor-measure and investigator-measure studies. However, 6 vendor-measure studies included data from more than 100 health systems, while just 1 investigator-measure study did.

### Differences in study aims and design

We coded study aims using the 3 aims of EHR log research identified in the prior review (Supplementary eTable 3).^11^ Aims were not mutually exclusive so each study could have more than 1 aim. While all vendor-measure studies *measured an amount of EHR use* (e.g., volume or duration),^2–4^^,^^6^^,^^19^^,^^24–58^ a third (33%) of investigator-measure studies did not,^60^^,^^68^^,^^74^^,^^78^^,^^80^^,^^82–87^^,^^89^^,^^91^^,^^92^^,^^98^^,^^102^^,^^109^^,^^112^^,^^115^ focusing instead on the other 2 aims (*P* < .001). Vendor-measure studies were more likely to *characterize EHR or clinical workflows*, such as when EHR activities were performed during the day (68% vs 43% of articles, *P* = .016), while investigator-measure studies were more likely to *characterize team dynamics*, for example, using record coaccess to determine which clinicians routinely worked together (23% vs 3% of studies, *P* = .004).

Most studies were observational (91 articles) while a minority were experimental (11 articles). Nine of the observational studies examined associations between EHR use and an outcome in the cohort of observed users such as burnout,^6^^,^^7^^,^^33^^,^^46^^,^^47^^,^^67^^,^^69^ turnover,^28^ and stress.^71^ Three observational studies examined associations between EHR use and clinical outcomes including next-day discharge,^78^ length of stay,^84^ and speed of consult response.^97^ Three observational studies predicted patient-provider relationships based on EHR use.^82^^,^^98^^,^^103^ Ten observational studies were longitudinal, comparing EHR use at different points in time including before and after the start of the coronavirus disease 2019 (COVID-19) pandemic,^2^^,^^3^^,^^34^^,^^37^^,^^89^ before and after a change to the EHR interface,^107^ before and after policy changes,^101^^,^^109^ and at different points of clinical training.^32^^,^^52^ The 11 experimental studies examined the impact of scribes,^30^^,^^41^^,^^111^ EHR training,^38^^,^^45^^,^^54^^,^^119^ and interface changes on EHR use.^75^^,^^93^^,^^94^^,^^113^ Only one of the experimental studies was a randomized controlled trial.^75^ Across both observational and experimental studies, 41 articles compared EHR use across different groups of users including comparisons by specialty (14 studies),^4^^,^^7^^,^^25^^,^^29^^,^^31^^,^^36^^,^^42^^,^^48^^,^^51^^,^^58^^,^^59^^,^^85^^,^^105^^,^^115^ clinical role (12),^24^^,^^26^^,^^33^^,^^37^^,^^46^^,^^65^^,^^76^^,^^95^^,^^99^^,^^106^^,^^108^^,^^115^ gender (8),^19^^,^^34^^,^^47^^,^^49^^,^^66^^,^^68^^,^^70^^,^^106^ year in residency (8),^31^^,^^32^^,^^35^^,^^39^^,^^52^^,^^56^^,^^57^^,^^95^ organization (3),^24^^,^^44^^,^^75^ and country (1).^27^ Vendor-measure studies were more likely than investigator-measure studies to make such comparisons of EHR use by user group (65% vs 25% of studies, *P* < .001).

### Differences in study measures

The prior review of EHR audit log research identified 5 general measures that can be derived from EHR logs.^11^ These included counts of specific actions or activities, durations of time, recurring sequences of actions, clusters of similar users or actions, and networks of users. We identified 2 significant differences in how these general measures were employed. First, while all vendor-measure studies reported a duration of time, just half of investigator-measure studies did (100% vs 48% of studies, *P* < .001). Second, no vendor-measure study created clinician networks while 9 investigator-measure studies did (0% vs 15% of studies, *P* = .011).

**Figure 4.**
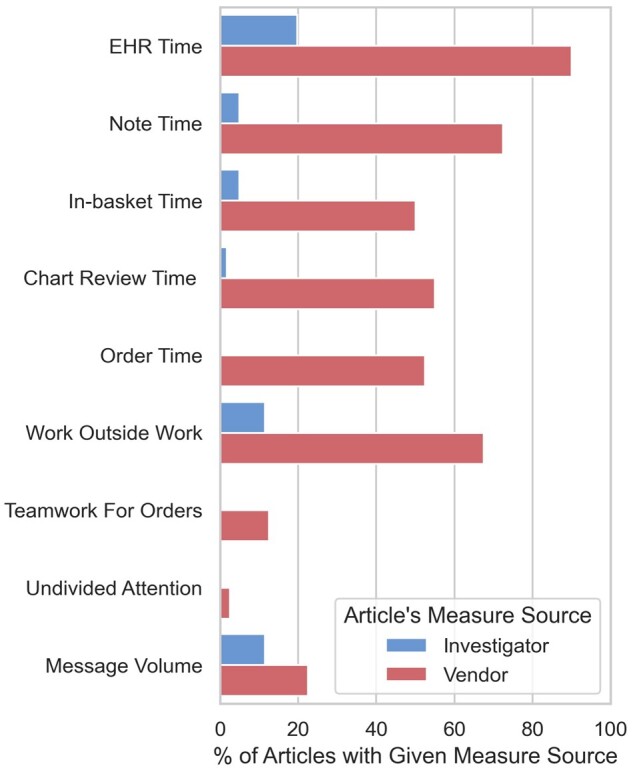
Articles reporting select measures, by source of measures reported in article.

Looking at specific measures of EHR use reported in each study reveals additional differences, particularly regarding durations of EHR use (Figure 4). Reported time-based measures included total time in the EHR (49 articles),^3^^,^^4^^,^^19^^,^^24–33^^,^^35–49^^,^^51–59^^,^^61^^,^^66^^,^^69–73^^,^^77^^,^^79^^,^^95^^,^^98^ time in notes (32 articles),^3^^,^^4^^,^^7^^,^^19^^,^^24–32^^,^^35–38^^,^^40–46^^,^^48^^,^^51^^,^^55–58^^,^^79^^,^^109^ time in inbox (23 articles),^2–4^^,^^19^^,^^24^^,^^25^^,^^27–29^^,^^35–38^^,^^42^^,^^43^^,^^45^^,^^47^^,^^48^^,^^51^^,^^61^^,^^62^^,^^70^^,^^71^ time in chart review (23 articles),^3^^,^^4^^,^^24–27^^,^^29^^,^^31^^,^^32^^,^^35^^,^^36^^,^^40^^,^^42^^,^^44–46^^,^^48^^,^^51^^,^^55^^,^^56^^,^^58^^,^^79^ time in orders (21 articles),^3^^,^^4^^,^^19^^,^^24–27^^,^^29^^,^^31^^,^^32^^,^^35^^,^^36^^,^^40^^,^^44^^,^^45^^,^^48^^,^^51^^,^^55–58^ and time outside of normal working hours (35 articles).^3^^,^^4^^,^^6^^,^^19^^,^^25^^,^^27–30^^,^^32^^,^^34^^,^^36–44^^,^^47–51^^,^^53^^,^^55^^,^^66^^,^^67^^,^^69–72^^,^^77^^,^^119^ Vendor-measure studies were more likely than investigator-measure studies to report each of these 6 time-based measures (*P* < .001 in each case). While all vendor-measure studies reported at least 1 duration of active EHR use (e.g., EHR time, inbox time), just 28% of investigator-measure studies did so, with the remainder reporting specific measures related to counts of EHR actions (e.g., number of records opened, number of searches performed), the structure of clinical teams (e.g., betweenness, centrality), or the duration of clinical events (e.g., exam length, duration of shift).

Definitions of what constituted EHR use outside normal working hours varied (Table 1). Twenty-two studies reported a measure based on a set time period, of which 7 unique periods were used (5:30 pm–8:30 am and 12:30 pm–1:30 pm, 5:30 pm–7 am, 6 pm–6 am, 6 pm–7 am, 7 pm–7 am, 7 pm–8 am, and 7:30 pm–7:30 am).^4^^,^^6^^,^^19^^,^^25^^,^^27^^,^^29^^,^^32^^,^^34^^,^^36^^,^^39^^,^^40^^,^^42^^,^^48^^,^^49^^,^^51^^,^^53^^,^^55^^,^^66^^,^^69–71^ An overlapping set of 22 articles, including 9 that also reported time period-based measures, reported at least 1 measure based on clinician schedules.^4^^,^^6^^,^^19^^,^^27^^,^^28^^,^^30^^,^^34^^,^^37^^,^^38^^,^^40^^,^^41^^,^^43^^,^^44^^,^^47–50^^,^^67^^,^^69^^,^^72^^,^^77^^,^^119^ These included time outside scheduled hours on days with appointments, time on days without appointments, and time after the patient checked out. These schedule-based measures differed in whether they (1) measured active EHR use or all time logged into the EHR, (2) included mid-day meetings or breaks in scheduled hours, or (3) included the 30 or 60 min before and after the first and last appointment of the day in scheduled hours.

**Table 1.** Number of studies reporting various measures of EHR work outside of work

	Measure	Studies reporting
Vendor-derived measures	Cerner’s after hours^a^	7
	Epic’s time outside scheduled hours^b^	6
	Epic’s time on unscheduled days	6
	Epic’s 7 pm—7 am	6
	Epic’s pajama time^c^	5
	Epic’s time outside scheduled hours ^+^ Epic’s time on unscheduled days	3
Investigator-derived measures	All time outside scheduled clinic time	5
	All time after patient check-out	2
	5:30 pm—8:30 am and 12:30 pm—1:30 pm^d^	2
	7 pm—8 am^d^	1
	6 pm—7 am^d^	1
	7:30 pm—7:30 am^d^	1

a Six pm—6 am on weekdays and all time on weekends.

b Time before 30 min prior to first appointment and after 30 min after last appointment of the day on days with scheduled appointments.

c 5:30 pm—7 am on weekdays and all time on weekends outside scheduled appointments.

d Including all time on weekends.

EHR: electronic health record.

A variety of denominators were used to normalize measures of EHR time. The most frequent denominator was days (31 articles) including days in a reporting period, days with a scheduled appointment/shift, weekdays, or weekend days/holidays.^2–4^^,^^24^^,^^26^^,^^27^^,^^31^^,^^34–36^^,^^38^^,^^40^^,^^41^^,^^43–46^^,^^48–50^^,^^52^^,^^54^^,^^55^^,^^59^^,^^62^^,^^66^^,^^69–71^^,^^79^^,^^95^ The next most popular denominators were number of appointments (12 articles),^24^^,^^25^^,^^29^^,^^30^^,^^32^^,^^35^^,^^38^^,^^40^^,^^42^^,^^44^^,^^72^^,^^77^ and patients (9 articles).^26^^,^^31^^,^^39^^,^^41^^,^^51^^,^^53^^,^^56^^,^^57^^,^^98^ Eleven other denominators were also used including EHR time per hour of clinic, per 8 h of clinic, per clinic session, per shift, per week, per month, per quarter, per year, per procedure, per note, and per residency.

Five articles measured teamwork for orders,^19^^,^^28^^,^^38^^,^^40^^,^^51^ and 1 measured undivided attention.^37^ Finally, 16 articles reported EHR inbox message volume. Vendor-measure studies were more likely to report teamwork for orders (13% vs 0%, *P* = .008), but there were no significant differences in the reporting of undivided attention or message volume, though the number of studies reporting either measure was low.

### Differences in study methods

Two decisions analysts make when creating time-based measures from EHR logs are (1) how to determine when a user is actively using the EHR and (2) how to map individual actions, such as clicking on an information panel, to activities such as inbox management or chart review. We review how these methods were reported and validated.

While 70 articles measured a duration of time,^2–4^^,^^6^^,^^7^^,^^19^^,^^24–62^^,^^66–73^^,^^76^^,^^77^^,^^79–81^^,^^83^^,^^92–96^^,^^98^^,^^102^^,^^108^^,^^109^^,^^111^^,^^119^ 12 of these measured a duration between 2 specific points in time (e.g., duration of an appointment, or shift),^60^^,^^68^^,^^76^^,^^80^^,^^81^^,^^83^^,^^92–94^^,^^96^^,^^102^^,^^111^ leaving 58 articles which measured a duration of active EHR use.^2–4^^,^^6^^,^^7^^,^^19^^,^^24–59^^,^^61^^,^^62^^,^^66^^,^^67^^,^^69–73^^,^^77^^,^^79^^,^^95^^,^^98^^,^^108^^,^^109^^,^^119^ Of these 58 articles, 34 described (or referenced an article that described) how active use was defined.^3^^,^^4^^,^^19^^,^^24–29^^,^^31^^,^^32^^,^^35^^,^^39^^,^^41^^,^^42^^,^^44^^,^^47^^,^^48^^,^^52^^,^^53^^,^^55–58^^,^^62^^,^^66^^,^^69^^,^^72^^,^^73^^,^^77^^,^^79^^,^^95^^,^^98^^,^^108^ There was no difference in reporting between vendor-measure or investigator-measure studies (59% vs 60% of relevant articles, *P* = 1.000), but there were differences in method. The vendor-measure studies all used Epic’s 5-s threshold to identify periods of inactivity or Cerner’s method of defining active use as either actions occurring less than 45 seconds apart or performing more than 15 keystrokes, 3 mouse clicks, or 1700 pixels of mouse movement in a minute. Investigator-measure studies used a wider range of methods to determine active EHR use including timeouts for inactivity (i.e., 30 s, 45 s, 1 min, 90 s, 5 min) and looking for any activity in 1- or 5-min blocks of time.

These differences in how active EHR use was defined, combined with differences in how measures were normalized, limit comparison of EHR times across studies, especially studies that rely on investigator-derived measures (Table 2). While 17 investigator-measure studies reported at least 1 measure of EHR time, in only 3 instances (involving 5 unique studies) did a pair of studies use both the same method of determining active EHR use and the same denominator. Of the 40 vendor-measure studies that reported a duration of active EHR use, 39 shared their method of determining active EHR use and measure denominator with at least one other study. For example, 17 studies reported durations of EHR use per day as tracked by Epic’s active use algorithm while 9 studies reported durations of EHR use per patient as tracked by Cerner’s active use algorithm.

**Table 2.** Number of studies reporting a duration of active EHR use by method of determining active use and measure denominator

		Measure denominator				
		Day	Ambulatory patients^a^	Calendar time^b^	Hours of patient contact^c^	Inpatient patients^d^
Method of determining active EHR use for vendor-derived measures	Epic's algorithm^e^	17	6	5	2	
	Cerner's algorithm^f^	5	9	5	2	2
	Unstated	1	2			
Method of determining active EHR use for investigator-derived measures	<30-s pause		1			
	<45-s pause	1				
	<1-min pause	1				
	<90-s pause	2		2	1	
	<5-min pause					1
	Any activity in 1-min period		2			
	Any activity in 5-min period	1				
	Unstated	3	1		2	

a Includes per patient, appointment, and note.

b Includes per week, month, quarter, year, and residency.

c Includes per hour, 8 h, clinic-session, and shift.

d Includes per patient, note, and procedure.

e Active EHR use defined as periods with <5-s gaps between clicks or keystrokes.

f Active EHR use defined as actions occurring less than 45 seconds apart or greater than 15 keystrokes, 3 mouse clicks, or 1700 pixels of mouse movement per minute.

EHR: electronic health record.

Of the 37 articles that reported durations of EHR use for specific activities such as inbox management or chart review,^2–4^^,^^7^^,^^19^^,^^24–32^^,^^35–38^^,^^40–48^^,^^51^^,^^55–58^^,^^62^^,^^70^^,^^71^^,^^79^^,^^109^ 16 described how actions were mapped to activities, either by providing a list of actions mapped to each activity, or by stating that the EHR vendor had done the mapping.^2–4^^,^^7^^,^^25^^,^^27^^,^^29^^,^^32^^,^^40^^,^^43^^,^^44^^,^^57^^,^^58^^,^^62^^,^^70^^,^^71^ There was no difference in reporting between vendor-measure or investigator-measure studies (67% vs 39% of relevant articles, *P* = .370).

Eight articles reported the results of measure validation, with no difference in reporting between vendor-measure and investigator-measure studies (10% vs 5%, *P* = .473).^6^^,^^53^^,^^79^^,^^80^^,^^83^^,^^85^^,^^102^^,^^103^ These articles compared resident duty hours derived from EHR logs with self-reported or Global Positioning System tracked hours (3 articles),^80^^,^^83^^,^^102^ patient-clinician relationships derived from logs with those from COVID-19 contact tracers or self-report (2 articles),^85^^,^^103^ EHR time derived from logs with self-report (2 articles),^6^^,^^53^ and EHR login time derived from logs with those observed through screen recording (1 article).^79^ Five additional articles referenced previous validations for measures of EHR time including articles referencing Epic’s,^28^ Cerner’s,^29^ Arndt et al’s,^66^^,^^124^ and “other EHR vendors” methods.^68^ The article referencing Cerner’s validation described comparing measures derived from EHR audit logs with those from direct observation of 337 clinicians across 5 health systems.^29^ However, the results of this validation have not been published.

### Limitations of EHR event log research

Reviewed articles mentioned several limitations of EHR log research (Supplementary eTable 4). Three of the most frequently mentioned limitations echo those observed in the prior review of audit log research^11^: EHR logs do not provide a full view of clinical activity which can involve physical and digital interactions outside the EHR (22 articles),^3^^,^^26^^,^^34^^,^^43^^,^^55^^,^^57^^,^^58^^,^^61^^,^^65^^,^^68^^,^^73^^,^^74^^,^^84–86^^,^^89^^,^^91^^,^^92^^,^^95^^,^^103^^,^^109^^,^^112^ qualitative methods are needed to better understand the context and motivation for observed work (15 articles),^32^^,^^46^^,^^53^^,^^62^^,^^64^^,^^68^^,^^72^^,^^80^^,^^85^^,^^91^^,^^96^^,^^97^^,^^101^^,^^115^^,^^118^ and logs may not contain enough detail to observe complex workflows (13 articles).^4^^,^^26^^,^^32^^,^^43^^,^^51^^,^^56^^,^^59^^,^^69^^,^^75^^,^^81^^,^^89^^,^^90^^,^^99^

Reviewed articles also raised 8 new limitations not identified in the prior review. Four of these limitations related to measure accuracy and granularity, each of which was mentioned in both investigator-measure and vendor-measure studies with no difference in reporting rates (*P* > .05 in each case). Seventeen articles argued that current measures may systematically underestimate EHR use,^2^^,^^4^^,^^6^^,^^26^^,^^27^^,^^43^^,^^48^^,^^57^^,^^61^^,^^62^^,^^70^^,^^73^^,^^79^^,^^80^^,^^83^^,^^88^^,^^119^ for example, undercounting time on activities such as chart review which may involve periods of reading without clicks or keystrokes. Alternatively, 6 articles suggested current methods may overestimate EHR use, often referring to being unable to distinguish ambulatory and inpatient EHR use or in-clinic and at-home use.^24^^,^^59^^,^^67^^,^^80^^,^^88^^,^^119^ Eight articles cited a need to validate measures of EHR use,^31^^,^^42^^,^^51^^,^^53^^,^^55^^,^^60^^,^^68^^,^^98^ while 9 mentioned needing additional quantitative data, most often scheduling data, to perform a finer-grained analysis, such as determining which activities were performed during or after clinic hours.^27^^,^^29^^,^^32^^,^^48^^,^^64^^,^^66^^,^^71^^,^^91^

Four additional limitations referenced measure interpretability and scope. While these limitations were mentioned in both vendor-measure and investigator-measure studies, the first 3 limitations were raised more often in vendor-measure studies (*P* < .05 in each case). Seven articles cited difficulties with interpreting proprietary vendor-derived metrics,^3^^,^^6^^,^^28^^,^^32^^,^^38^^,^^42^^,^^78^ while another 7 articles argued that current definitions of work outside of work do not match actual schedules in the observed setting.^6^^,^^27^^,^^29^^,^^32^^,^^37^^,^^44^^,^^55^ Ten articles highlighted how current measures track the work of physicians and advanced-practice providers, but not other team members such as nurses or scribes.^3^^,^^4^^,^^24^^,^^27^^,^^33^^,^^35^^,^^39^^,^^44^^,^^82^^,^^109^ Finally, 6 articles cited missing data from inpatient settings or being unable to distinguish between inpatient and outpatient work for clinicians working across both settings.^3^^,^^4^^,^^27^^,^^47^^,^^90^^,^^119^

## DISCUSSION

### Key findings

The number of studies using event logs to observe EHR use continues to grow. While researchers continue to derive their own measures of EHR use from event logs, a growing body of literature relies on measures derived by EHR vendors. Vendor- and investigator-derived measures are used independently—only one study in this review employed both—and for different kinds of research. Studies employing vendor-derived measures focused almost exclusively on ambulatory physicians and APPs while studies employing investigator-derived measures examined EHR use in both ambulatory and acute care by a broader range of users (e.g., physicians, APPs, nurses, technicians, students, scribes). Studies employing vendor-derived measures were more likely to report durations of EHR use while those employing investigator-derived measures were more likely to examine communication and collaboration in care teams. While most studies reported a duration of active EHR use (e.g., EHR time), variation in how active use was defined and how measures were normalized (e.g., by day, appointment, hour) limit comparison across studies, especially studies employing investigator-derived measures. And while all studies based on event logs share a common set of strengths and limitations, studies employing vendor-derived measures were more likely to raise concerns about measure opacity (though all measures developed by someone other than the investigator may be viewed as opaque), misalignment with typical clinic schedules, and measure availability for certain clinical roles.

### Strengths and limitations

This scoping review updates a prior review of audit log research and expands it to include all research based on EHR event logs,^11^ providing insight into the increasing use of vendor-derived measures in research. This review also has several limitations which future work could address. First, it considered measures derived from EHR event logs but excluded studies based on related data such as logs from other health information technology (e.g., telephone logs), and timestamps stored in patient records (e.g., check-in time). Research analyzing these data may have distinct aims, measures, and methods compared to the literature surveyed in this review. Second, to align our methods with those employed in the prior review, we limited our search to articles indexed in PubMed which may have excluded relevant articles published in engineering or social science venues. We minimized this risk by performing forward and backward reference searches. Third, article abstraction is a subjective process which, in this review, was largely performed by a single author. We mitigated potential bias by using a coding scheme derived from a prior review, and by iteratively revising and validating the coding scheme through independent coding of the same articles by 2 authors.

### Implications and future directions

Vendor-provided measures remove many of the barriers to conducting log-based research but are limited in scope. The reviewed studies only reported vendor-derived measures for physicians and advanced practiced providers, and most of these only measured EHR use in ambulatory settings. However, the work of nurses, medical assistants, students, technicians, scribes, and other team members—many of whom experience significant documentation burden—should not be overlooked.^125^ While some vendors provide measures of EHR use for these roles, the lack of studies reporting them suggests a lack of measure awareness or accessibility. Until vendor-provided measures of EHR use are more widely available and accessible for all EHR users, investigators will need to continue deriving custom measures for some users.

Vendor-provided measures automate the process of turning strings of logged events into durations of EHR use. However, vendor-provided measures are largely lacking for constructs such as workflow and teamwork. Given the strong association between EHR time outside scheduled hours and physician burnout,^6–9^ a closer examination of the relationship between who does what EHR work, when, and with whom may provide additional insight into the sources of documentation burden and burnout. Investigators should continue to create measures of these important but complex topics of teamwork and workflow, and work with vendors to operationalize them for broader use.

While widely reported, durations of active EHR use (e.g., EHR time) are variously defined, which limits synthesis of evidence on critical topics such as documentation burden and its link to burnout. This was particularly true of studies that employed investigator-derived measures, most of which had unique definitions of active EHR use. Vendor-derived measures enable greater consistency across studies, though different vendors use different methods of defining and normalizing EHR time, making cross-vendor comparison difficult.^19^ Some variations are needed to ensure measures are appropriate for their domain of use (e.g., inpatient vs outpatient care). However, much current measurement variation—especially in the definitions of active EHR use, the normalization of time-based measures, and the definitions of “work outside work”—is unnecessary, hinders comparison, and reflects separate groups creating their own measures from scratch. The research community should continue to develop and adopt standardized measures of EHR use, such as the 7 measures of ambulatory EHR use proposed by a national research network of EHR log researchers,^23^ and to work with vendors to shape vendor-derived measures as they become de facto standards. Researchers might also assist the synthesis of evidence across studies by more clearly reporting their methods, including in online repositories and supplemental material, and by reporting conversion factors, such as the average number of appointments per day, to enable measures to be converted from one denominator to another.

More work is also needed to ensure measure validity.^126^ Studies should clearly demonstrate the *criterion validity* of measures they employ (i.e., that values derived from event logs match those from gold-standard methods such as direct observation), as well as *content validity* (i.e., that measures include all relevant EHR activity) and *construct validity* (i.e., that measures relate to a construct/theory/trait of interest such as documentation burden). The number of unique definitions of EHR time outside scheduled hours (Table 1) demonstrates the difficulty of operationalizing the construct of work outside work. Inbox management has likewise proved difficult to define in a way that captures all inbox work. Vendor-derived measures only count time spent interacting with dedicated inbox screens, while some investigators, recognizing that inbox management often involves visiting other parts of the EHR and work outside of the EHR, have included all time between opening and responding to a message.^70^^,^^71^ This distinction reveals the gaps that emerge when equating interface time with activity time.

Vendors and investigators have unique roles to play in measure development. EHR vendors are well positioned to curate generalizable measures of the duration and volume of EHR activity agnostic to specific workflows. Some vendors have provided the methodological decisions behind their measures to customers or referenced them in studies,^13^^,^^29^ but these methods are inconsistently reported in the studies that depend on them. For example, only 59% of the studies which reported a duration of active EHR use described how active use was defined. Publishing vendor’s validation studies, which have been referenced in several studies but not explicitly reported, would also help ensure accurate accounting of log-based measures. Investigators in turn are uniquely positioned to validate the measures they derive from event logs, particularly those of workflow and team dynamics which may be workflow or site-specific. Ideally, validation efforts by both vendors and investigators will include explorations of whether measures are equally valid across different strata of clinicians (e.g., part time vs full time; with scribes vs without scribes), particularly for those measures that rely on heuristics to define concepts such as active use, or typical clinic schedules.

## CONCLUSION

EHR event logs are an increasingly vital source of data for research. This updated scoping review demonstrates the continued growth of event log research, particularly research employing vendor-derived measures of EHR use. This growth is welcome as more groups investigate the sources of documentation burden and links between EHR use and clinician well-being. For this research to provide the strongest evidence to inform policy and practice, more work is needed to develop, standardize, and validate log-based measures of EHR use.

## FUNDING

NCA is supported by a training grant from the Agency for Healthcare Research and Quality (T32-HS026116-04). Dr ERM is supported by grants and contracts from the National Institute on Drug Abuse, American Medical Association, and Agency for Healthcare Research and Quality unrelated to this work. Funding for open access publishing was provided by the Sarah M. Pritchard Faculty Support Fund.

## AUTHOR CONTRIBUTIONS

AR and NCA contributed to the research design, data analysis, and manuscript preparation. ERM contributed to the research design and manuscript preparation.

## SUPPLEMENTARY MATERIAL

Supplementary material is available at *Journal of the American Medical Informatics Association* online.

## Supplementary Material

ocac177_Supplementary_Data

## Data Availability

No new data were generated or analyzed in support of this research.
