# Correction to: Using event logs to observe interactions with electronic health records: an updated scoping review shows increasing use of vendor-derived measures

**DOI:** 10.1093/jamia/ocac224

**Published:** 2022-11-15

This is a correction to: Adam Rule, Edward R Melnick, Nate C Apathy, Using event logs to observe interactions with electronic health records: an updated scoping review shows increasing use of vendor-derived measures, Journal of the American Medical Informatics Association, 2022;, ocac177, https://doi.org/10.1093/jamia/ocac177

In the originally published version of this manuscript, there was one error and two omissions:

First, the manuscript reported that 35 articles were published in the 2-1/2 years before July 2019 that used audit logs to observe electronic health record (EHR) use. This number should have been 33 articles.

Figure 2 has been replaced with an updated Figure which reduces the number of audit log articles published in 2019 by two articles.

Second, the manuscript described Cerner’s definition of active EHR use as performing more than 15 keystrokes, 3 mouse clicks, or 1700 pixels of mouse movement in a minute. This is partially correct: Cerner's reported method also defines active EHR use as periods where logged events occur less than 45 seconds apart.

Finally, the manuscript omitted the following acknowledgement: “Funding for open access publishing was provided by the Sarah M. Pritchard Faculty Support Fund.”

These errors have been corrected online.

