# Supplementary material for: Using event logs to observe interactions with electronic health records: an updated scoping review shows increasing use of vendor-derived measures
Source: J Am Med Inform Assoc. 2022 Sep 29;30(1):144–54. doi: 10.1093/jamia/ocac177 (PMC9748581; doi:10.1093/jamia/ocac177)
Supplement: ocac177_Supplementary_Data [file ocac177_supplementary_data.docx]

**Using Event Logs to Observe Interactions with Electronic Health Records: An Updated Scoping Review Shows Increasing Use of Vendor-Derived Measures**

Adam Rule, PhD, Edward R. Melnick, MD MHS, Nate C. Apathy, PhD

**Supplemental Material**

*Table of Contents*

eMethods

eTable1: Article details

eTable2: Article scope by source of data

eTable3: Article aims, measures, and methods by data source.

eTable4: Study limitations

**eMethods**

PubMed Search Query (last search, January 10, 2022):

((“audit”[All Fields] AND (log[All Fields] OR file*[All Fields] OR data[All Fields]))

OR (log[All Fields] AND (file*[All Fields] OR "event"[All Fields] OR "access"[All Fields] OR "system"[All Fields] OR "usage"[All Fields] OR "activity"[All Fields]))

OR "metadata"[All Fields] OR "meta data"[All Fields] OR "meta-data"[All Fields]

OR "timestamp"[All Fields]

OR "interaction patterns"[All Fields]

OR "utilization patterns"[All Fields]

OR "ehr us*"[All Fields]

OR "ehr activity"[All Fields]

OR "electronic health record us*"[All Fields]

OR "electronic health record activity"[All Fields]

OR "ehr time"[All Fields]

OR "ehr burden"[All Fields]

OR "electronic health record time"[All Fields]

OR "electronic health record burden"[All Fields]

OR "documentation time"[All Fields]

OR "documentation burden"[All Fields]

OR "inbox"[All Fields]

OR "inbasket"[All Fields]

OR "in-basket"[All Fields]

OR "electronic messag*"[All Fields]

OR "note length"[All Fields]

OR ("note"[All Fields] AND ("source"[All Fields] OR "attribution"[All Fields]))

OR "chart clos*"[All Fields]

OR "closed same day"[All Fields]

OR "closed on the same day"[All Fields])

AND ((("computerised"[All Fields] OR "computerized"[All Fields] OR "electronic"[All Fields]) AND ("health"[All Fields] OR "medical"[All Fields]) AND ("record"[All Fields] OR "records"[All Fields])) OR ("electronic health records"[MeSH Terms]) OR (“medical records systems, computerized"[MeSH Terms]))

Inclusion Criteria:

We included articles which met the following criteria:

1. peer-reviewed
2. reports original research (e.g., not a review or commentary)
3. is a conference or journal article (e.g., not an abstract, poster, or case study)
4. analyzes EHR use rather than use of a related technology such as a patient portal, health information exchange, or mobile health app
5. analyzes EHR logs rather than primary EHR data
6. if analyzing audit logs, involves secondary use of audit logs rather than use of audit logs for their primary purpose of access control
7. does not study interactions with clinical decision support
8. written in English

eTable 1: Article details

| Article | | | | Participants | | | | Data | | | | Aims | | | General Measures | | | | | Specific Measures | | | | | | | | | Methods | | |
| --- | --- | --- | --- | --- | --- | --- | --- | --- | --- | --- | --- | --- | --- | --- | --- | --- | --- | --- | --- | --- | --- | --- | --- | --- | --- | --- | --- | --- | --- | --- | --- |
| Ref | PMID | First Author | Year | Users | Specialty | Domain | Multi-Institution | EHR | Scope | Investigator-Measure | Vendor -Measures | EHR Use | Workflow | Team | Action Count | Time Duration | Sequence | Cluster | Network | EHR Time | Note Time | Inbox Time | Chart Review Time | Orders Time | Work Outside Work | Teamwork for Orders | Undivided Attention | Message Volume | Define Active Use | Action-Activity Map | Validation |
| 2 | 34636917 | Nath B | 2021 | Physicians | All | Ambulatory |  | Epic | Feature |  | X | X |  |  | X | X |  |  |  |  |  | X |  |  |  |  |  | X |  | X |  |
| 3 | 34888680 | Holmgren AJ | 2021 | Physicians, APPs | All | Ambulatory | X | Epic | EHR |  | X | X | X |  | X | X |  |  |  | X | X | X | X | X | X |  |  | X | X | X |  |
| 4 | 33749732 | Rotenstein LS | 2021 | Physicians, APPs | All | Ambulatory | X | Epic | EHR |  | X | X | X |  | X | X |  |  |  | X | X | X | X | X | X |  |  | X | X | X |  |
| 6 | 32016375 | Adler-Milstein J | 2020 | Physicians | Primary Care | Ambulatory |  | Epic | EHR |  | X | X | X |  | X | X |  |  |  |  |  |  |  |  | X |  |  | X |  |  | X |
| 19 | 33822970 | Melnick ER | 2021 | Physicians | All | Ambulatory | X | Epic; Cerner | EHR |  | X | X | X |  | X | X |  |  |  | X | X | X |  | X | X | X |  |  | X |  |  |
| 24 | 32876342 | Giliberto JP | 2021 | Physicians; APPs | Surgical | Ambulatory | X | Epic | EHR |  | X | X |  |  |  | X |  |  |  | X | X | X | X | X |  |  |  |  | X |  |  |
| 25 | 33139456 | Overhage JM | 2020 | Physicians | Pediatric | Ambulatory | X | Cerner | EHR |  | X | X | X |  |  | X |  |  |  | X | X | X | X | X | X |  |  |  | X | X |  |
| 26 | 32740334 | Watson MD | 2020 | Residents, APPs | All | Ambulatory |  | Cerner | EHR |  | X | X |  |  | X | X |  |  |  | X | X |  | X | X |  |  |  |  | X |  |  |
| 27 | 33315048 | Holmgren AJ | 2021 | Physicians, APPs | All | Ambulatory | X | Epic | EHR |  | X | X | X |  | X | X |  |  |  | X | X | X | X | X | X |  |  | X | X | X |  |
| 28 | 34636911 | Melnick ER | 2021 | Physicians | All | Ambulatory |  | Epic | EHR |  | X | X | X |  |  | X |  |  |  | X | X | X |  |  | X | X |  |  | X |  |  |
| 29 | 31931523 | Overhage JM | 2020 | Physicians | All | Ambulatory | X | Cerner | EHR |  | X | X | X |  |  | X |  |  |  | X | X | X | X | X | X |  |  |  | X | X |  |
| 30 | 33683446 | Lam C | 2021 | Physicians | Surgical | Ambulatory |  |  | EHR |  | X | X | X |  |  | X |  |  |  | X | X |  |  |  | X |  |  |  |  |  |  |
| 31 | 32305335 | Maloney SR | 2020 | Residents | Surgical | Ambulatory |  | Cerner | EHR |  | X | X |  |  |  | X |  |  |  | X | X |  | X | X |  |  |  |  | X |  |  |
| 32 | 34348409 | Holmgren AJ | 2021 | Residents | All | Ambulatory |  | Cerner | EHR |  | X | X | X |  | X | X |  |  |  | X | X |  | X | X | X |  |  |  | X | X |  |
| 33 | 32719859 | Hilliard RW | 2020 | Physicians, APPs | All | Ambulatory | X | Epic | EHR |  | X | X |  |  | X | X |  |  |  | X |  |  |  |  |  |  |  | X |  |  |  |
| 34 | 33600728 | Beiser M | 2021 | Physicians | All | Ambulatory |  | Epic | EHR |  | X | X | X |  |  | X |  |  |  |  |  |  |  |  | X |  |  |  |  |  |  |
| 35 | 34667838 | Wandell GM | 2021 | Physicians | Surgical | Ambulatory |  | Epic | EHR |  | X | X |  | X |  | X |  |  |  | X | X | X | X | X |  |  |  |  | X |  |  |
| 36 | 34613950 | Kesler K | 2021 | Physicians | All | Ambulatory |  | Epic | EHR |  | X | X | X |  | X | X |  |  |  | X | X | X | X | X | X |  |  | X |  |  |  |
| 37 | 34261172 | Moore C | 2021 | Physicians, APPs | All | Ambulatory |  | Cerner | EHR |  | X | X | X |  |  | X |  |  |  | X | X | X |  |  | X |  | X |  |  |  |  |
| 38 | 33575787 | Hollister-Meadows L | 2021 | Physicians | All | Ambulatory |  | Epic | EHR |  | X | X | X |  | X | X |  |  |  | X | X | X |  |  | X | X |  |  |  |  |  |
| 39 | 32050270 | Anderson J | 2020 | Physicians | Primary Care | Ambulatory |  | Cerner | EHR |  | X | X | X |  |  | X |  |  |  | X |  |  |  |  | X |  |  |  | X |  |  |
| 40 | 32074650 | Baxter SL | 2020 | Physicians | Surgical | Ambulatory |  | Epic | EHR |  | X | X | X |  | X | X |  |  |  | X | X |  | X | X | X | X |  |  |  | X |  |
| 41 | 34020102 | Jhaveri P | 2021 | Physicians | Primary Care | Ambulatory |  | Cerner | EHR |  | X | X | X |  |  | X |  |  |  | X | X |  |  |  | X |  |  |  | X |  |  |
| 42 | 34713161 | Khairat S | 2021 | Physicians | Pediatric | Ambulatory |  | Epic | EHR |  | X | X |  |  |  | X |  |  |  | X | X | X | X |  | X |  |  |  | X |  |  |
| 43 | 34897493 | Nguyen OT | 2021 | Physicians | Primary Care | Ambulatory |  | Epic | EHR |  | X | X | X |  | X | X |  |  |  | X | X | X |  |  | X |  |  |  |  | X |  |
| 44 | 32525047 | Baxter SL | 2021 | Physicians | Surgical | Ambulatory | X | Epic | EHR |  | X | X | X |  |  | X |  |  |  | X | X | X | X | X | X |  |  |  | X | X |  |
| 45 | 34435176 | Sieja A | 2021 | Physicians | Medical | Ambulatory |  | Epic | EHR |  | X | X |  |  |  | X |  |  |  | X | X | X | X | X |  |  |  |  |  |  |  |
| 46 | 34545781 | Mosquera MJ | 2021 | Physicians | Medical | Ambulatory |  |  | EHR |  | X | X | X |  |  | X |  |  |  | X | X |  | X |  |  |  |  |  |  |  |  |
| 47 | 33877310 | McPeek-Hinz E | 2021 | Physicians, APPs | All | Ambulatory |  | Epic | EHR |  | X | X | X |  | X | X |  |  |  | X |  | X |  |  | X |  |  | X | X |  |  |
| 48 | 34241631 | Rotenstein LS | 2021 | Physicians, APPs | All | Ambulatory | X | Epic | EHR |  | X | X | X |  | X | X |  |  |  | X | X | X | X | X | X |  |  | X | X |  |  |
| 49 | 33284311 | Tait SD | 2021 | Physicians | All | Ambulatory |  | Epic | EHR |  | X | X | X |  |  | X |  |  |  | X |  |  |  |  | X |  |  |  |  |  |  |
| 50 | 31073856 | Saag HS | 2019 | Physicians | All | Ambulatory |  | Epic | EHR |  | X | X | X |  |  | X |  |  |  |  |  |  |  |  | X |  |  |  |  |  |  |
| 51 |  | Wilkinson K | 2021 | Physicians | All | Ambulatory |  | Cerner | EHR |  | X | X | X |  | X | X |  |  |  | X | X | X | X | X | X | X |  |  |  |  |  |
| 52 | 32609757 | Verma G | 2020 | Physicians | All | Acute |  | Cerner | EHR |  | X | X |  |  |  | X |  |  |  | X |  |  |  |  |  |  |  |  | X |  |  |
| 53 | 32673234 | Tajirian T | 2020 | Physicians | Medical | Acute |  |  | EHR |  | X | X | X |  |  | X |  |  |  | X |  |  |  |  | X |  |  |  | X |  | X |
| 54 | 33029643 | Simpson JR | 2021 | Residents, APPs | Surgical | Acute |  | Epic | EHR |  | X | X |  |  |  | X |  |  |  | X |  |  |  |  |  |  |  |  |  |  |  |
| 55 | 32215894 | Krawiec C | 2020 | Physicians | Pediatric | Acute |  | Cerner | EHR |  | X | X | X |  |  | X |  |  |  | X | X |  | X | X | X |  |  |  | X |  |  |
| 56 | 34693185 | Olson E | 2021 | Residents | Medical | Acute |  | Cerner | EHR |  | X | X |  |  | X | X |  |  |  | X | X |  | X | X |  |  |  |  | X |  |  |
| 57 | 32654998 | Watson MD | 2020 | Residents | Surgical | Both |  | Cerner | EHR |  | X | X |  |  |  | X |  |  |  | X | X |  | X | X |  |  |  |  | X | X |  |
| 58 | 34933572 | Loszko A | 2021 | Physicians | Surgical | Both |  | Cerner | EHR |  | X | X |  |  | X | X |  |  |  | X | X |  | X | X |  |  |  |  | X | X |  |
| 7 | 31260371 | Tai-Seale M | 2019 | Physicians | All | Ambulatory |  | Epic | Feature | X |  | X |  |  |  | X |  |  |  |  | X |  |  |  |  |  |  | X |  | X |  |
| 59 | 33483138 | Cox ML | 2021 | Physicians | Surgical | Ambulatory |  | Epic | EHR | X |  | X |  |  |  | X |  |  |  | X |  |  |  |  |  |  |  |  |  |  |  |
| 60 | 33301282 | Neprash HT | 2021 | Physicians | Primary Care | Ambulatory | X | athenahealth | Feature | X |  |  | X |  |  | X |  |  |  |  |  |  |  |  |  |  |  |  |  |  |  |
| 61 | 33274310 | Long CP | 2020 | Residents | Surgical | Ambulatory |  | Epic | EHR | X |  | X |  |  | X | X |  |  |  | X |  |  |  |  |  |  |  |  |  |  |  |
| 62 | 33475754 | Lieu TA | 2021 | Physicians | Primary Care | Ambulatory |  | Epic | Feature | X |  | X | X |  | X | X |  |  |  |  |  | X |  |  |  |  |  | X | X | X |  |
| 63 | 31627022 | Perros I | 2020 | Physicians, APPs | Primary Care | Ambulatory |  | Epic | EHR | X |  | X | X |  |  |  |  | X |  |  |  |  |  |  |  |  |  |  |  |  |  |
| 64 | 31183688 | Cohen GR | 2019 | Physicians | Primary Care | Ambulatory | X |  | EHR | X |  | X |  |  | X |  |  |  |  |  |  |  |  |  |  |  |  |  |  |  |  |
| 65 | 34345803 | Chen JS | 2021 | Physicians | Surgical | Ambulatory |  | Epic | Feature | X |  | X |  |  | X |  |  |  |  |  |  |  |  |  |  |  |  |  |  |  |  |
| 66 | 31573912 | Attipoe S | 2019 | Physicians | Primary Care | Ambulatory |  | Epic | EHR | X |  | X | X |  |  | X |  |  |  | X |  |  |  |  | X |  |  |  | X |  |  |
| 67 | 34261173 | Sinha A | 2021 | Physicians | Medical | Ambulatory |  | Epic | EHR | X |  | X | X |  |  | X |  |  |  |  |  |  |  |  | X |  |  |  |  |  |  |
| 68 | 32997909 | Ganguli I | 2020 | Physicians | Primary Care | Ambulatory | X | athenahealth | EHR | X |  |  | X |  |  | X |  |  |  |  |  |  |  |  |  |  |  |  |  |  |  |
| 69 |  | Micek M | 2020 | Physicians | Primary Care | Ambulatory |  | Epic | EHR | X |  | X | X |  |  | X |  |  |  | X |  |  |  |  | X |  |  |  | X |  |  |
| 70 | 33063087 | Akbar F | 2021 | Physicians | Primary Care | Ambulatory |  | Epic | Feature | X |  | X | X |  | X | X |  |  |  | X |  | X |  |  | X |  |  | X |  | X |  |
| 71 | 33908888 | Akbar F | 2021 | Physicians | Primary Care | Ambulatory |  | Epic | Feature | X |  | X | X |  | X | X |  | X |  | X |  | X |  |  | X |  |  |  |  | X |  |
| 72 | 33936431 | Hribar MR | 2021 | Physicians | Surgical | Ambulatory |  | Epic | Feature | X |  | X | X |  |  | X |  |  |  | X |  |  |  |  | X |  |  |  | X |  |  |
| 73 | 33618957 | Mohler SA | 2021 | All | Surgical | Ambulatory |  | Epic | EHR | X |  | X |  |  |  | X |  |  |  | X |  |  |  |  |  |  |  |  | X |  |  |
| 74 | 31160011 | Zhu X | 2019 | All | All | Ambulatory |  | Cerner | Feature | X |  |  |  | X |  |  |  |  | X |  |  |  |  |  |  |  |  |  |  |  |  |
| 75 | 32414376 | Hatch B | 2020 | All | All | Ambulatory | X | Epic | Feature | X |  | X |  |  | X |  |  |  |  |  |  |  |  |  |  |  |  |  |  |  |  |
| 76 | 34528233 | Steitz BD | 2021 | All | All | Ambulatory |  |  | Feature | X |  | X | X |  | X | X |  |  |  |  |  |  |  |  |  |  |  | X |  |  |  |
| 77 | 35059685 | Dusek HL | 2021 | Physicians, Scribes | Surgical | Ambulatory |  | Epic | EHR | X |  | X | X | X |  | X |  |  |  | X |  |  |  |  | X |  |  |  | X |  |  |
| 78 | 34592753 | Zhang X | 2021 | Physicians | All | Acute |  | Epic | EHR | X |  |  | X |  | X |  |  |  |  |  |  |  |  |  |  |  |  |  |  |  |  |
| 79 | 34380167 | Sinha A | 2021 | Physicians, APPs | Pediatric | Acute |  | Epic | EHR | X |  | X |  |  |  | X |  |  |  | X | X |  | X |  |  |  |  |  | X |  | X |
| 80 | 33929943 | Soleimani H | 2021 | Residents | Medical | Acute |  | Epic | EHR | X |  |  | X |  |  | X |  |  |  |  |  |  |  |  |  |  |  |  |  |  | X |
| 81 | 34963142 | Gong JJ | 2021 | Residents | Medical | Acute |  | Epic | Feature | X |  | X | X |  | X | X |  | X |  |  |  |  |  |  |  |  |  |  |  |  |  |
| 82 | 31460936 | Schumacher DJ | 2019 | Residents | Medical | Acute |  | Epic | EHR | X |  |  |  | X | X |  |  |  |  |  |  |  |  |  |  |  |  |  |  |  |  |
| 83 | 31830096 | Dziorny AC | 2019 | Residents | Pediatric | Acute |  | Epic | EHR | X |  |  | X |  |  | X |  |  |  |  |  |  |  |  |  |  |  |  |  |  | X |
| 84 | 32637942 | Kim C | 2019 | All | Pediatric | Acute |  |  | EHR | X |  |  |  | X |  |  |  |  | X |  |  |  |  |  |  |  |  |  |  |  |  |
| 85 | 34477566 | Li P | 2021 | All | Pediatric | Acute |  | Epic | EHR | X |  |  | X | X |  |  |  |  | X |  |  |  |  |  |  |  |  |  |  |  | X |
| 86 | 32170716 | Chen Y | 2019 | All | Pediatric | Acute |  | StarPanel | EHR | X |  |  |  | X | X |  |  |  | X |  |  |  |  |  |  |  |  |  |  |  |  |
| 87 | 33621187 | Yan C | 2021 | All | Medical | Acute |  |  | EHR | X |  |  |  | X |  |  |  |  | X |  |  |  |  |  |  |  |  |  |  |  |  |
| 88 | 34549294 | Wang M | 2021 | All | All | Acute |  | Epic | Feature | X |  | X | X |  | X |  |  |  |  |  |  |  |  |  |  |  |  |  |  |  |  |
| 89 | 34637393 | Mannering H | 2021 | All | Pediatric | Acute |  |  | EHR | X |  |  |  | X |  |  |  |  | X |  |  |  |  |  |  |  |  |  |  |  |  |
| 90 | 34612825 | Payne TH | 2021 | All | Medical | Acute |  | Cerner | Feature | X |  | X |  |  | X |  |  |  |  |  |  |  |  |  |  |  |  |  |  |  |  |
| 91 | 33576432 | Chen B | 2021 | Nurses | Pediatric | Acute |  | Epic | EHR | X |  |  | X |  |  |  |  | X |  |  |  |  |  |  |  |  |  |  |  |  |  |
| 92 | 30632467 | Despins LA | 2019 | Nurses | Medical | Acute |  | Cerner | EHR | X |  |  | X |  |  | X |  |  |  |  |  |  |  |  |  |  |  |  |  |  |  |
| 93 | 33845323 | Horn JJ | 2021 | Nurses | Pediatric | Acute |  | Cerner | Feature | X |  | X |  |  | X | X |  |  |  |  |  |  |  |  |  |  |  |  |  |  |  |
| 94 | 32643778 | Sutton DE | 2020 | Nurses | All | Acute | X | Cerner | Feature | X |  | X |  |  | X | X |  |  |  |  |  |  |  |  |  |  |  |  |  |  |  |
| 95 | 31419265 | Chi J | 2019 | Physicians, Students | Medical | Acute |  | Epic | EHR | X |  | X |  |  | X | X |  |  |  | X |  |  |  |  |  |  |  |  | X |  |  |
| 96 | 33320098 | Kim S | 2020 | Residents | All | Both |  | DARWIN | EHR | X |  | X |  |  | X | X |  |  |  |  |  |  |  |  |  |  |  |  |  |  |  |
| 97 | 32130157 | Jung KY | 2020 | Physicians | All | Both |  | DARWIN | EHR | X |  | X |  |  |  |  |  |  |  |  |  |  |  |  |  |  |  |  |  |  |  |
| 98 | 32583389 | Mai MV | 2020 | Residents | Pediatric | Both |  | Epic | EHR | X |  |  |  | X | X | X |  |  |  | X |  |  |  |  |  |  |  |  | X |  |  |
| 99 | 33936435 | Jones B | 2021 | Physicians | Pediatric | Both |  | Epic | EHR | X |  | X |  |  | X |  |  | X |  |  |  |  |  |  |  |  |  |  |  |  |  |
| 100 | 31423117 | Gellert G | 2019 | Physicians | All | Both |  |  | Feature | X |  | X |  |  | X |  |  |  |  |  |  |  |  |  |  |  |  |  |  |  |  |
| 101 | 34661667 | Steitz BD | 2021 | Physicians | All | Both |  |  | Feature | X |  | X | X |  | X |  |  |  |  |  |  |  |  |  |  |  |  |  |  |  |  |
| 102 | 34507910 | Lin JA | 2021 | Residents | Surgical | Both |  | Epic | EHR | X |  |  | X |  |  | X |  |  |  |  |  |  |  |  |  |  |  |  |  |  | X |
| 103 | 34623426 | Hong P | 2021 | All | All | All |  |  | EHR | X |  |  |  | X | X |  |  |  |  |  |  |  |  |  |  |  |  |  |  |  | X |
| 104 | 31730181 | Kawamoto K | 2019 | All | Pediatric | Both |  | Epic | Feature | X |  | X |  |  | X |  |  |  |  |  |  |  |  |  |  |  |  |  |  |  |  |
| 105 | 32142128 | Ruppel H | 2020 | All | All | Both |  | Epic | Feature | X |  | X | X |  | X |  |  |  | X |  |  |  |  |  |  |  |  |  |  |  |  |
| 106 | 32557441 | Sulieman L | 2020 | All | All | Both |  |  | Feature | X |  | X |  |  | X |  |  |  |  |  |  |  |  |  |  |  |  |  |  |  |  |
| 107 | 32134449 | Adler-Milstein J | 2020 | All | All | Both |  | Epic | Feature | X |  | X |  |  | X |  |  |  |  |  |  |  |  |  |  |  |  |  |  |  |  |
| 108 | 33709066 | Nestor JG | 2021 | All | All | Both |  | Allscripts | Feature | X |  | X | X |  | X | X |  | X |  |  |  |  |  |  |  |  |  |  | X |  |  |
| 109 | 34233368 | Stevens LA | 2021 | Physicians, Students | Pediatric | Both |  |  | Feature | X |  |  | X |  | X | X |  |  |  |  | X |  |  |  |  |  |  |  |  |  |  |
| 110 | 31811860 | Henriksen BS | 2020 | Physicians | Surgical | Ambulatory |  | Epic | Feature | X |  | X |  |  |  |  |  |  |  |  |  |  |  |  |  |  |  |  |  |  |  |
| 111 | 33452084 | Zallman L | 2021 | Physicians | Primary Care | Ambulatory |  | Epic | Feature | X |  | X | X |  |  | X |  |  |  |  |  |  |  |  |  |  |  |  |  |  |  |
| 112 | 31438036 | Steitz BD | 2019 | Physicians | All | Ambulatory |  |  | Feature | X |  |  |  | X | X |  |  |  | X |  |  |  |  |  |  |  |  |  |  |  |  |
| 113 | 31438405 | Chu L | 2019 | Physicians | Primary Care | Ambulatory |  | Epic | Feature | X |  | X |  |  | X |  |  |  |  |  |  |  |  |  |  |  |  | X |  |  |  |
| 114 | 34757419 | Lutz MK | 2021 | Physicians | All | Ambulatory |  | Epic | Feature | X |  | X |  |  | X |  |  |  |  |  |  |  |  |  |  |  |  | X |  |  |  |
| 115 | 31682267 | Steitz BD | 2020 | All | All | Ambulatory |  |  | Feature | X |  |  |  | X | X |  |  |  | X |  |  |  |  |  |  |  |  | X |  |  |  |
| 116 | 34279650 | Rule A | 2021 | All | All | Ambulatory |  | Epic | Feature | X |  | X |  |  |  |  |  |  |  |  |  |  |  |  |  |  |  |  |  |  |  |
| 117 | 34664655 | Rule A | 2021 | All | All | Ambulatory |  | Epic | Feature | X |  | X |  | X | X |  |  |  |  |  |  |  |  |  |  |  |  |  |  |  |  |
| 118 | 33629079 | Rule A | 2020 | All | Surgical | Ambulatory |  | Epic | Feature | X |  | X |  | X | X |  |  |  |  |  |  |  |  |  |  |  |  |  |  |  |  |
| 119 | 31984357 | DiAngi YT | 2019 | Physicians | Pediatric | Ambulatory |  | Epic | EHR | X | X | X | X |  |  | X |  |  |  |  |  |  |  |  | X |  |  |  |  |  |  |

eTable 2: Article scope by source of data

|  |  | All Studies | | Investigator Measure | | Vendor Measure | | Investigator vs. Vendor Measure^a^ | | |
| --- | --- | --- | --- | --- | --- | --- | --- | --- | --- | --- |
|  |  | N | % | N | % | N | % | OR | 95% CI | p-value |
|  | Articles | 102 | 100 | 61 | 100% | 40 | 100% | - | - | - |
| Setting | Ambulatory | 63 | 62 | 29 | 48 | 33 | 83 | - | - | 0.002 |
|  | Acute (Inpatient and Emergency) | 23 | 23 | 18 | 30 | 5 | 13 |  |  |  |
|  | Both | 16 | 16 | 14 | 23 | 2 | 5 |  |  |  |
| EHR Users | All Physicians | 50 | 49 | 23 | 38 | 26 | 65 | - | - | <0.001 |
|  | Residents / Fellows | 12 | 12 | 8 | 13 | 4 | 10 |  |  |  |
|  | Nurses | 4 | 4 | 4 | 7 | 0 | 0 |  |  |  |
|  | APPs | 1 | 1 | 0 | 0 | 1 | 3 |  |  |  |
|  | Physicians and APPs | 11 | 11 | 2 | 3 | 9 | 23 |  |  |  |
|  | Physicians and Students | 2 | 2 | 2 | 3 | 0 | 0 |  |  |  |
|  | Physicians and Scribes | 1 | 1 | 1 | 2 | 0 | 0 |  |  |  |
|  | All Users | 21 | 21 | 21 | 34 | 0 | 0 |  |  |  |
| Institution | Single | 87 | 85 | 56 | 92 | 30 | 75 | 3.681 | 1.033, 15.056 | 0.025 |
|  | Multiple | 15 | 15 | 5 | 8 | 10 | 25 |  |  |  |
| Activity Scope | All EHR Activity | 69 | 68 | 29 | 48 | 39 | 98 | 0.024 | 0.001, 0.161 | <0.001 |
|  | Specific Activity | 33 | 32 | 32 | 52 | 1 | 3 |  |  |  |
| Specialty | All | 41 | 40 | 21 | 34 | 20 | 50 | - | - | 0.217 |
|  | Surgical | 18 | 18 | 9 | 15 | 9 | 23 |  |  |  |
|  | Pediatric | 16 | 16 | 12 | 20 | 3 | 8 |  |  |  |
|  | Primary Care | 15 | 15 | 11 | 18 | 4 | 10 |  |  |  |
|  | Medical | 12 | 12 | 8 | 13 | 4 | 10 |  |  |  |
| EHR | Vendor | 84 | 82 | 46 | 75 | 37 | 93 | - | - | 0.084 |
|  | Locally-developed | 3 | 3 | 3 | 5 | 0 | 0 |  |  |  |
|  | Not Stated | 15 | 15 | 12 | 20 | 3 | 8 |  |  |  |

^a^ p-values and odds ratio confidence intervals are from Fisher’s Exact Test comparing distributions between studies analyzing investigator-derived and vendor-derived measures. APPs = Advanced Practice Providers

eTable 3: Article aims, measures, and methods by data source

|  |  | All Studies | | Investigator Measure | | Vendor Measure | | Investigator vs. Vendor Measure^a^ | | |
| --- | --- | --- | --- | --- | --- | --- | --- | --- | --- | --- |
|  |  | N | % | N | % | N | % | OR | 95% CI | p-value |
| Articles |  | 102 | 100 | 61 | 100 | 40 | 100 | - | - | - |
| Aims | EHR Use | 82 | 80 | 41 | 67 | 40 | 100 | 0.000 | 0.000, 0.229 | <0.001 |
|  | Workflow | 54 | 53 | 26 | 43 | 27 | 68 | 0.361 | 0.142, 0.887 | 0.016 |
|  | Team | 15 | 15 | 14 | 23 | 1 | 3 | 7.336 | 1.997, 41.421 | 0.004 |
| Design | Experimental | 11 | 11 | 5 | 8 | 5 | 13 | 0.628 | 0.134, 2.942 | 0.515 |
|  | Subgroup Comparison | 41 | 40 | 15 | 25 | 26 | 65 | 0.179 | 0.067, 0.456 | <0.001 |
| Measures (General) | Action Count | 53 | 52 | 35 | 57 | 18 | 45 | 1.637 | 0.683, 3.973 | 0.308 |
|  | Time Duration | 70 | 69 | 29 | 48 | 40 | 100 | 0.000 | 0.000, 0.100 | <0.001 |
|  | Action Sequence | 0 | 0 | 0 | 0 | 0 | 0 | 0.000 | 0.000, Inf | 1.000 |
|  | Cluster | 6 | 6 | 6 | 10 | 0 | 0 | Inf | 0.801, Inf | 0.079 |
|  | Network | 9 | 9 | 9 | 15 | 0 | 0 | Inf | 1.406, Inf | 0.011 |
| Measures (Specific) | EHR Time | 48 | 47 | 12 | 20 | 36 | 90 | 0.029 | 0.006, 0.100 | <0.001 |
|  | Note Time | 32 | 31 | 3 | 5 | 29 | 73 | 0.021 | 0.003, 0.083 | <0.001 |
|  | In-basket Time | 23 | 23 | 3 | 5 | 20 | 50 | 0.054 | 0.009, 0.207 | <0.001 |
|  | Chart Review Time | 23 | 23 | 1 | 2 | 22 | 55 | 0.014 | 0.000, 0.100 | <0.001 |
|  | Order Time | 21 | 21 | 0 | 0 | 21 | 53 | 0.000 | 0.000, 0.069 | <0.001 |
|  | Work Outside Work | 35 | 34 | 7 | 11 | 27 | 68 | 0.065 | 0.019, 0.192 | <0.001 |
|  | Teamwork For Orders | 5 | 5 | 0 | 0 | 5 | 13 | 0.000 | 0.000, 0.674 | 0.008 |
|  | Undivided Attention | 1 | 1 | 0 | 0 | 1 | 3 | 0.000 | 0.000, 25.574 | 0.396 |
|  | Message Volume | 16 | 16 | 7 | 11 | 9 | 23 | 0.450 | 0.129, 1.511 | 0.168 |
| Methods | Define Active Use | 24 | 60^b^ | 10 | 59^b^ | 24 | 60^b^ | 0.953 | 0.261, 3.618 | 1.000 |
|  | Action-Activity Map | 16 | 43^c^ | 4 | 67^c^ | 12 | 39^c^ | 3.067 | 0.373, 38.882 | 0.370 |
|  | Validate Measure | 8 | 8 | 6 | 10 | 2 | 5 | 2.059 | 0.344, 21.921 | 0.473 |

^a^ p-values and odds ratio confidence intervals are from Fisher’s Exact Test comparing distributions between studies using investigator-derived and vendor-derived measures.

^b^ Percent of articles which measured a duration active EHR use

^c^ Percent of articles which measured a duration of EHR use for a particular activity such as orders, notes, in-basket, or chart review.

eTable 4: Study limitations

|  | All Studies | | Investigator Measure | | Vendor Measure | | Investigator vs. Vendor Measure^a^ | | |
| --- | --- | --- | --- | --- | --- | --- | --- | --- | --- |
|  | N | % | N | % | N | % | OR | 95% CI | p-value |
| Articles | 102 | 100 | 61 | 100 | 40 | 100 | - | - |  |
| Missing Non-EHR Activity | 22 | 22 | 15 | 25 | 7 | 18 | 1.531 | 0.516, 4.958 | 0.466 |
| Missing Qualitative Insight | 15 | 15 | 12 | 20 | 3 | 8 | 2.990 | 0.733, 17.693 | 0.151 |
| Logs/Measures Too Course | 13 | 13 | 7 | 11 | 6 | 15 | 0.736 | 0.193, 2.897 | 0.763 |
| Measures Underestimate Time | 17 | 17 | 8 | 13 | 8 | 20 | 0.607 | 0.179, 2.057 | 0.409 |
| Measures Overestimate Time | 6 | 6 | 4 | 7 | 1 | 3 | 2.713 | 0.256, 138.088 | 0.646 |
| Need Non-Log Data | 9 | 9 | 4 | 7 | 5 | 13 | 0.494 | 0.092, 2.470 | 0.477 |
| Measures Need Validation | 8 | 8 | 3 | 5 | 5 | 13 | 0.366 | 0.054, 2.013 | 0.259 |
| Measures are Proprietary | 7 | 7 | 1 | 2 | 6 | 15 | 0.096 | 0.002, 0.846 | 0.015 |
| WOW Definition | 7 | 7 | 0 | 0 | 7 | 18 | 0.000 | 0.000, 0.408 | 0.001 |
| Missing Clinical Roles | 10 | 10 | 2 | 3 | 8 | 20 | 0.138 | 0.014, 0.750 | 0.013 |
| Missing Acute Care | 6 | 6 | 1 | 2 | 4 | 10 | 0.153 | 0.003, 1.621 | 0.079 |

^a^ p-values and odds ratio confidence intervals are from Fisher’s Exact Test comparing distributions between studies using EHR Logs and Vendor-derived Measures.
